# Chemometrics-Driven Variability Evaluation of Phenolic Composition, Antioxidant Capacity, and α-Glucosidase Inhibition of *Sorbus aucuparia* L. Fruits from Poland: Identification of Variability Markers for Plant Material Valorization

**DOI:** 10.3390/antiox12111967

**Published:** 2023-11-05

**Authors:** Magdalena Rutkowska, Aleksandra Owczarek-Januszkiewicz, Anna Magiera, Mateusz Gieleta, Monika A. Olszewska

**Affiliations:** Department of Pharmacognosy, Faculty of Pharmacy, Medical University of Lodz, 1 Muszynskiego St., 90-151 Lodz, Poland; magdalena.rutkowska@umed.lodz.pl (M.R.); aleksandra.owczarek@umed.lodz.pl (A.O.-J.); anna.magiera@umed.lodz.pl (A.M.); mateusz.gieleta@stud.umed.lodz.pl (M.G.)

**Keywords:** *Sorbus aucuparia*, fruits, variability, polyphenols, antioxidant activity, hydroxyl radical scavenging, antidiabetic activity, α-glucosidase

## Abstract

*Sorbus aucuparia* L. (rowan tree) is a widely distributed European plant, valued for its nutritional and medicinal qualities. The medicinal application of rowanberries, relying particularly on their antioxidant and antidiabetic effects, is closely connected with the presence of numerous phenolic compounds. However, the broad geographical occurrence of rowan trees may contribute to fluctuations in fruit composition, influencing their biological properties. This study aimed to identify the constituents most involved in this variability to facilitate effective quality control. The investigation encompassed 20 samples collected from diverse locations across Poland, evaluated in terms of the variation in composition and bioactivity. The UHPLC-PDA-ESI-MS^n^ study identified 45 different constituents, including flavonoids, phenolic acid and flavon-3-ols. The detected compounds were quantitatively assessed by HPLC-PDA, alongside spectrophotometric evaluation of total phenolic content and the content of high-molecular-weight proanthocyanidins (TPA). Additionally, ^•^OH scavenging capacity and α-glucosidase inhibition were included as bioactivity parameters. Chemometric analyses, including hierarchical cluster analysis and principal component analysis, revealed geographically dependent variability, with low to moderate variation observed for most factors (variation coefficients 20.44–44.97%), except for flavonoids (variation coefficients 45–76%). They also enabled the selection of seven constituents and TPA as the key markers of variability and biological activity of rowanberries. These markers could be employed for quality control of the fruits, offering a more efficient and cost-effective approach compared to full phytochemical analysis.

## 1. Introduction

*Sorbus aucuparia* L. (mountain-ash, rowan tree) is a deciduous plant that is widely distributed throughout most of Europe, ranging from Iceland, Scandinavia, and Russia to Spain, Italy, and the Balkans. It may also be found in northern and southwestern Asia. Additionally, it has been introduced to Canada and the United States as an ornamental tree [1]. It thrives in such diverse geographical regions due to its high tolerance to a short vegetative season and various environmental stressors such as high summer temperatures, drought, cold and frost. The species, appreciated for its decorative, nutritional and medicinal properties, has become popular in both cultivation and in its natural settings [2,3].

*S. aucuparia* L. provides edible fruits used for preparation of juices, jams, jellies, alcoholic beverages, confectionary, etc. [4]. They are also utilized as a traditional herbal remedy for the prevention and treatment of several conditions, such as diabetes, atherosclerosis, or hypertension [2]. Based on existing research, their biological mechanisms primarily include antioxidant, antiradical, and enzyme-inhibitory activity [5,6,7,8,9,10,11], which are attributed to the presence of a rich fraction of various polyphenolic compounds, i.e., proanthocyanidins, flavonols, and pseudodepsides of caffeic and ferulic acids [5,6,7]. However, the data gathered thus far on the qualitative and quantitative composition of the rowan fruits reported by different authors have been contradictory [5,10,12,13,14,15,16,17]. Therefore, the matter of compositional variability has been brought to light.

As previously reported, the variability in the profile of the rowanberry constituents might result from genetic factors, i.e., the typical of *Sorbus* spp. tendency for apomixes [18]. On the other hand, considering the well-established role of secondary metabolites in plant adaptive mechanisms and defense against biotic and abiotic stressors, the composition of many plant species is highly influenced by their geographical location [19,20,21]. This is especially true for species inhabiting regions with significantly different environmental conditions, and it may also apply to rowanberries and plant materials obtained thereof.

Such susceptibility to changes under the influence of environmental conditions pose significant challenges in the quality control analyses, which are crucial step to ensure the safety and effectiveness of plant materials [20,22]. One of the initial steps in establishing criteria for quality control of new plant materials is the identification of suitable analytical markers. While the markers of quality and biological activity often comprise the main active constituents, we must also consider the importance of minor metabolites. These minor metabolites, due to their unique properties and potential synergistic relationships, may prove to be key chemical markers [22]. On the other hands, it is essential to keep the number of controlled parameters as limited as possible to ensure the simplicity of routine quality control analyses. 

Given the above mentioned premises, this study aimed to address several important questions: (I) To what extent can the qualitative and quantitative variability be anticipated in rowanberries? (II) How do these variations influence the biological properties of the fruits? (III) What quality control measures should be implemented for fruit products to ensure their value and potential functional applications? To achieve this goal, we conducted the first-ever assessment of the qualitative and quantitative variability in the phenolic profile of rowanberries collected from various regions of Poland, spanning from seaside to piedmont areas. This evaluation was performed using a range of profiling techniques, including UHPLC-PDA-ESI-MS^n^, HPLC-PDA, and UV–vis spectrophotometry. Additionally, guided by prior research [5,6], we examined the pharmacological diversity among the samples by assessing two model activities that reflect the antiradical and antidiabetic potential of rowanberries. These activities encompassed hydroxyl radical scavenging and α-glucosidase inhibition. Subsequently, we conducted an intra-species analysis of the fruits using various chemometric tools, including hierarchical cluster analysis (HCA) and principal component analysis (PCA). This approach made it possible to determine the impact of individual phenolics variability on biological potential of rowanberries, and enabled to identify the parameters that are optimal for the effective quality control of *S. aucuparia* fruits. 

## 2. Materials and Methods

### 2.1. Plant Materials

Mature fruits of *Sorbus aucuparia* L. were harvested in September 2019, specifically within a two-week period from 6 September 2019 to 19 September 2019. The fruits were collected from 20 different locations in Poland (Table 1), from a few to over a dozen trees depending on the size of the population in a given location, and were sourced from plants growing in their natural habitat or within botanical gardens. Authentication was carried out by prof. M.A. Olszewska from the Department of Pharmacognosy, Medical University of Lodz, Poland. To adhere to the traditional preparation method, the plant material was subjected to freezing for 24 h and lyophilized prior to the analyses [23].

### 2.2. Extracts Preparation

The lyophilized fruit samples (5 g) were defatted by pre-extraction with chloroform (100 mL, 45 min). Subsequently, extracts were prepared via reflux extraction (80 °C) of the plant material (defatted pellet) using a methanol–water mixture (1:1, *v*/*v*, 1 × 100 mL × 30 min and 2 × 50 mL × 20 min). The extracts were dried through vacuum evaporation (Büchi rotary evaporator with chiller set at −10 °C), lyophilized, weighed, and stored in the refrigerator. Prior to analyses, the extract samples were dissolved to achieve the appropriate concentration. All quantitative results were calculated based on the dry weight (dw) of the fruits.

### 2.3. Qualitative Profiling

The qualitative UHPLC-PDA-ESI-MS^n^ analysis was conducted following the previously described method [5], employing the same equipment, procedure, and reagents. The samples were prepared to achieve the final concentration of the extract solution at 50 μg/mL.

### 2.4. Quantitative Profiling

The extracts were subjected to quantitative analysis, that included the assessment of their total phenolic contents (TPH) and the contents of phenolic fractions and individual compounds using a validated HPLC-PDA method and the same equipment as previously described [25]. The samples were prepared to achieve the final concentration of the extract solution at 50 μg/mL. The analytes were quantified depending on their PDA spectra as equivalents of HPLC-pure external standards (Sigma-Aldrich, St. Louis, MO, USA; Phytolab, Vestenbergsgreuth, Germany): isoquercitrin (IQ) and hyperoside (HY) for flavonoid monoglycosides; quercetin 3-*O*-*β*-sophoroside (SQ) and rutin (RT) for flavonoid diglycosides; chlorogenic acid (CHA) for monocaffeoylquinic acid isomers; cynarin for dicaffeoylquinic acid isomers; caffeic acid (CFA) for hydroxycinnamic acid derivatives other than mono- and dicaffeoylquinic acid isomers; and procyanidin B2 (PB2) for proanthocyanidins.

Additionally, the total phenolic contents (TPC) and the total proanthocyanidins contents (TPA) were quantified using the Folin–Ciocalteu and *n*-butanol-HCl methods, respectively, following previously established protocols [25]. TPC measurement involved the removal of reducing sugars by C18 SPE, as described by Rutkowska et al. [5]. Results of the TPC and TPA assays were expressed in gallic acid equivalents (mg GAE/g of fruits dw) and cyanidin chloride equivalents (mg CyE/g of fruits dw), respectively.

### 2.5. Radical Scavenging Activity

The scavenging activity of *S. aucuparia* fruits against hydroxyl radical (^•^OH) was evaluated using the spectrophotometric method developed by Fu et al. [26] with some modifications [27]. The levels of ^•^OH (generated through the Fenton reaction with 1.5 mM FeSO_4_ and 6 mM H_2_O_2_) before and after the interaction with the tested analytes were monitored in the presence of salicylic acid (20 mM). After 30 min incubation at 37 °C, the absorbance was measured at 520 nm. The results were presented as SC_50_ values, derived from concentration–scavenging curves (5 calibration points). For direct comparison, the results were expressed in terms of ascorbic acid (AA) equivalents per extract dw (µmol AA/mg dw) or AA equivalents per polyphenols weight (µmol AA/mg GAE).

### 2.6. α-Glucosidase Inhibitory Activity

The impact of *S. aucuparia* fruits on yeast α-glucosidase activity was evaluated using the spectrophotometric method described by Kim et al. [28] with some modifications. The tested samples underwent preincubation with α-glucosidase (0.43 U/mL) at 37 °C for 15 min. Subsequently, *p*-nitrophenyl glucopyranoside (0.2 mg/mL) was introduced as the reaction substrate, and the mixture was incubated for 30 min. The reaction was halted by the addition of Na_2_CO_3_ (0.2 M). The activity was quantified by measuring the released of yellow-colored *p*-nitrophenol at 405 nm. The results were presented as IC_50_ values, derived from concentration-inhibition curves (5 calibration points). For direct comparison, the results were expressed in acarbose (AR) equivalents per extract dw (µmol AR/mg dw) or in AR equivalents per polyphenols weight (µmol AR/mg GAE).

### 2.7. Statistical Analyses

The results were presented as the means ± standard deviation (SD) based on the specified number of experiments. Statistical significance regarding differences between mean values was determined using a one-way ANOVA, followed by the post hoc Tukey’s or Fisher’s LSD tests for multiple comparisons. To assess the relationship between variables, Pearson correlation coefficients were calculated. All quantification results were used for the hierarchical cluster analysis (HCA), and selected parameters for principal component analysis (PCA). The calculations were performed using Satistica PL version 13.3 software for Windows (StatSoft Inc., Krakow, Poland) or JMP Trial version 17 software for Windows (SAS Institute, Cary, NC, USA) with *p*-values less than 0.05 regarded as significant.

## 3. Results and Discussion

### 3.1. Qualitative Variability of the Phenolic Composition

**Figure 1:**
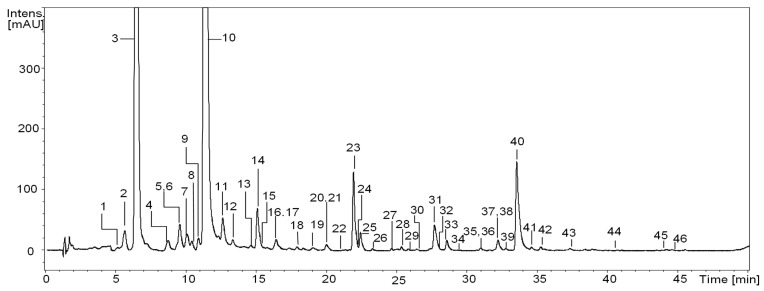
Representative UHPLC chromatogram (sample No. 17) at 325 nm of *S. aucuparia* fruits. Peak numbers refer to those implemented in Table 2.

The qualitative RP-LC-MS analysis of *S. aucuparia* fruits, sourced from various geographical regions of Poland (a total of twenty samples, as described in the Material and Methods section), resulted in the detection of forty-six constituents (UHPLC peaks 1–46; Figure 1, Table 2). Thirty-three of these constituents were found consistently across all samples. Identification of the analytes was achieved by comparing their UV–vis spectra, chromatographic behavior, and ESI-MS^n^ fragmentation patterns with reference compounds or information available in literature [10,12,13,14,15,16,29,30,31,32]. The identified compounds were categorized into several polyphenolic groups, including the most diverse hydroxycinnamic acid derivatives (with sixteen constituents), flavonols (with fourteen constituents), and proanthocyanidins (with ten constituents), as well as less numerous hydroxybenzoic acid derivatives (with two constituents) or flavonolignans (with three constituents). 

The most significant variability was observed within the group of flavonoids, particularly in derivatives of quercetin and kaempferol. Out of the fourteen detected flavonols, only five, namely quercetin 3-*O*-*β*-sophoroside (SQ, compound **23**), quercetin *O*-dihexoside (compound **25**), hyperoside (HY, compound **31**), rutin (RT, compound **32**), and isoquercitrin (IQ, compound **33**), were consistently present in all samples. Additionally, qualitative differences were noted in the groups of proanthocyanidins (compound **12** was found in only six samples, while compound **19** was present in sixteen samples) and flavonolignans (with compounds **30** and **39** being detected in eighteen and eleven samples, respectively). In contrast, phenolic acids appeared to exhibit qualitative stability, as representatives of this group were identified in all samples (Table 2).

The observed data confirm the existence of qualitative variability within the species, which may account for the conflicting data regarding *S. aucuparia* fruit composition reported by different authors [10,12,13,14,15,16,17]. For instance, Kylli et al. [10] identified RT, HY, IQ, quercetin dihexoside, hexoside-pentoside, and malonylglucoside in the 70% acetone-water extract from the fruits. In contrast, Gil-Izquierdo and Mellenthin [15] reported the presence of two additional kaempferol dihexosides and second quercetin dihexoside in the fruit juice, while quercetin malonylglucoside was not detected. 

### 3.2. Quantitative Variability of the Phenolic Composition

The next step in the analyses was to evaluate the range of quantitative variability in the investigated samples. 

**Figure 2:**
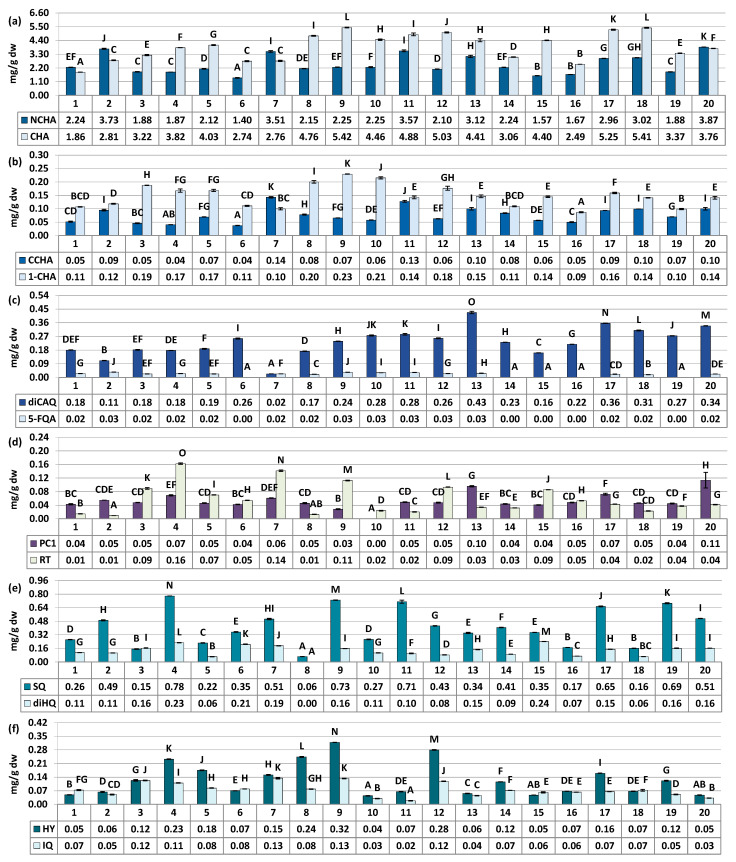
Quantitative profile of individual compounds in the *S. aucuparia* fruits (mg/g fruits dw). Results are presented as the means ± SD (*n* = 3). For each parameter, different superscript letters on particular charts (A–O) indicate significant differences (*p* < 0.05). (**a**) NCHA, 3-*O*-caffeoylquinic acid; CHA, 5-*O*-caffeoylquinic acid. (**b**) CCHA, 4-*O*-caffeoylquinic acid; 1-CHA, 1-*O*-caffeoylquinic acid. (**c**) diCAQA, 3,5-*O*-dicaffeoylquinic acid; 5-FQA, 5-ferulquinin acid. (**d**) PC1, procyanidin C1; RT, rutin. (**e**) SQ, quercetin 3-*O*-β-sophoroside; diHQ, quercetin *O*-dihexoside. (**f**) HY, hyperoside; IQ, isoquercitrin.

**Figure 3:**
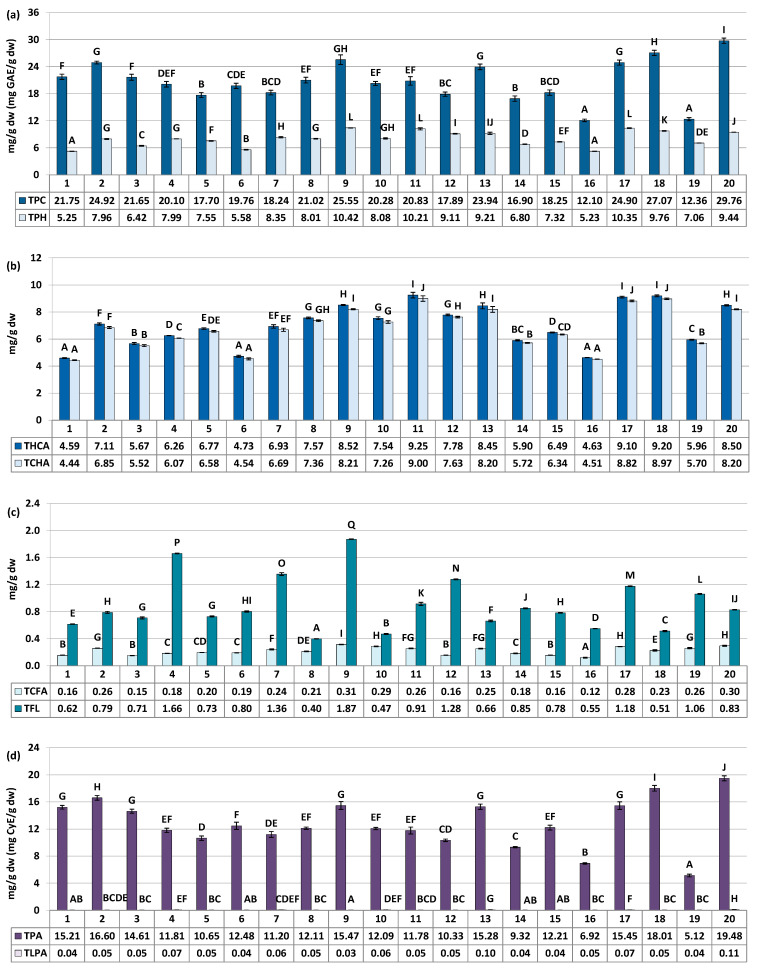
Quantitative profile of groups of compounds in the *S. aucuparia* fruits (mg/g fruits dw). Results are presented as the means ± SD (*n* = 3). For each parameter, different letters on particular charts (A–Q) indicate significant differences (*p* < 0.05). (**a**) TPC, total phenolic content in gallic acid equivalents (GAE) determined by the Folin–Ciocalteu assay; TPH, total phenolic content determined by RP-HPLC-PDA. (**b**) THCA, total content of hydroxycinnamic acid derivatives (TCHA + TCFA); TCHA, total content of mono- and dicaffeoylquinic acids isomers. (**c**) TCFA, total content of phenolic acids derivatives other than TCHA; TFL, total content of flavonoids. (**d**) TPA, total proanthocyanidin content in cyanidin chloride equivalents (CyE) determined by the *n*-butanol/HCl assay; TLPA, total content of proanthocyanidin determined by RP-HPLC-PDA.

The quantitative profile of the samples was assessed through UV–Vis spectrophotometry in terms of total contents of polyphenols (TPC, determined by the Folin–Ciocalteu assay) and proanthocyanidins (TPA, determined by the *n*-butanol/HCl assay). Additionally, the HPLC-PDA method was employed to quantify nineteen primary individual compounds that were within the limit of quantification, and calculate the total content of all polyphenols detected by HPLC (TPH), as well as the contents of specific groups of polyphenols (flavonoids, hydroxycinnamic acid derivatives, mono- and dicaffeoylquinic acid isomers, low-molecular-weight proanthocyanidins, etc.). Figure 2 illustrates the variability in the levels of twelve individual compounds predominant or specific to the phenolic profile of rowanberries (six phenolic acids, five flavonoids, and one proanthocyanidin). Figure 3 presents the variation in the total phenolics (TPC, TPH) and the contents of specific groups of polyphenols. For more detailed information see Supplementary Materials (Tables S1 and S2).

The major fruit constituents were mono- and dicaffeoylquinic acids (TCHA, 4.44–9.00 mg/g dw) and oligomeric and polymeric proanthocyanidins (TPA, 5.12–19.48 mg CyE/g dw) (Figure 2 and Figure 3). The TPC and TPH values determined using the Folin–Ciocalteu assay and HPLC-PDA, respectively, varied in a range of 12.10–29.76 mg GAE/g of fruits dw and 5.23–10.42 mg/g of fruits dw (Figure 3). These finding align with previous reports, which have indicated phenolic levels in rowanberries spanning from 5.25 mg GAE/g of fruits dw to 26.8 mg GAE/g of fruits dw, depending on sample origin and preparation [8,11,33]. Observed phenolic contents are also similar or higher than that of fruits of other *Sorbus* species, e.g., *S. aria* (3.9–29.8 mg GAE/g) or *S. domestica* (3.5–12.1 mg GAE/g) [11,33,34].

The variability of the samples in terms of total phenolic contents was relatively low, with coefficient of variation (CV) of 21.62% and 20.44% for the TPC and TPH levels, respectively (Table 3). However, when assessing quantitative differences in the contents of phenolic groups and individual compounds, the CVs increased significantly. They reached up to 43.59% for total flavonoids (TFL), and even higher values of 45.48%, 52.44%, 68.15%, and 75.87% were observed individually for IQ, SQ, HY, and RT, respectively. This pattern of variation, characterized by substantial differences in flavonoid quantities alongside lower variability in TPC, is consistent with findings previously reported for *S. aucuparia* and *S. aria* fruits collected from various locations in Serbia and Montenegro [33], as well as for *S. domestica* leaves colected from Poland and Croatia [35]. However, it is also worth to notice that flavonoids variability pattern seems to be closely species-related. For example, the content of RT in different populations of *Cornus mas* was about 2–20 µg/g of fresh berries, while for closely akin *Cornus sanguinea* it was about 2–120 µg/g [36]. The difference in flavonoids variability trend was also observed for fruits of black and red currant [37], as well as leaves and flowers of *Crataegus* ssp. [38], among others. Nevertheless, the results provide a further proof, that flavonoids seems to be the most changeable chemical group in rowan species, including *S. aucuparia* fruits. 

Observed variability can be attributed to various factors stemming from the multifaceted functions of flavonoids in plant physiology, particularly their role in the intricate interactions between plants and their environment [19,21,39]. For instance, they play a crucial role in safeguarding plant against herbivores, insects, nematodes, and pathogenic bacteria and fungi [7,29,30,31]. On the other hand, flavonoids can serve as attractants for pollinators, as well as birds or mammals, facilitating seed dispersal [7,32]. Moreover, plant secondary metabolites assume a critical role in the protection against abiotic stress conditions [19,20,21,39,40,41,42], among which access to light and solar radiation stands out as particularly significant factor for flavonoid biosynthesis in fruits [40]. For instance, the exposure to sunlight has been reported to induce the accumulation of flavonoids in various fruits, such as grape berries, cranberries, bilberries, raspberries, strawberries, pears, and apples. It has also been established, that UV and blue light exert the most pronounced effects on the expression of genes involved in the flavonoid biosynthesis. Within this group of compounds, flavonols, especially quercetin and kaempferol glycosides, appear to be the most responsive to environmental changes. Due to their specific chemical structure, characterized by the presence of an OH group in the C-3 position of the flavonoid skeleton and a double bond between C-2 and C-3 in the B-ring, flavonols are capable of selectively absorbing UV-B radiation. They also exhibit the ability to scavenge reactive oxygen species and mitigate photo-oxidative damage in plants [19,39]. The heightened sensitivity of flavonol biosynthesis to sunlight has been the subject of extensive research. For example, it has been shown that in grape berries exposed to shade, the accumulation of flavonols decreased significantly, in contrast to the less pronounced changes observed in other compounds tested, such as proanthocyanidins and anthocyanins [43]. Toxic heavy metals, salinity, water availability, and nutrient availability are among other environmental stress factors that influence the biosynthesis of different plant secondary metabolites [20,41,42]. 

However, in natural settings, plants are often exposed to multiple stressors simultaneously, making it challenging to predict the impact of all factors on the accumulation of specific compounds. Achieving such predictability would require tightly controlled breeding conditions, and even then, there would be still an element of uncertainty. This is because when two or more factors coincide, their effects can be additive, synergic, or one factor may take precedence [20]. 

Therefore, to ensure the quality of plant products, especially for functional applications, precise control over their composition is essential. This is particularly relevant for the active ingredients that primarily contribute to the desired biological properties. In the case of *S. aucuparia* fruits, various polyphenolic compounds present in complex fractions have been proposed to be responsible for their antioxidant and antidiabetic properties [5]. However, it remains to be determined, the variations in content of which polyphenols are the most significant in the context of fruits activity, and which might be considered irrelevant for the quality of rowanberries. To answer this question, we must first investigate to what extent the activity of rowanberries is subject to change.

### 3.3. The Variation in the Biological Activity Parameters

The ability of *S. aucuparia* fruit constituents to neutralize reactive oxygen species has been identified as a crucial mechanism contributing to the biological activity of rowanberries [5,9]. Among various radicals of physiological significance, the plant material demonstrated particularly strong affinity towards ^•^OH [5]. This is of paramount importance, since ^•^OH is one of the most highly reactive radical found in living organisms, capable of damaging various biomolecules and playing a role in the development of many cardiovascular and neurological disorders [44,45]. Secondly, the inhibitory activity of rowan fruits on carbohydrate-digesting enzymes, especially α-glucosidase [6,46], has been identified as an important mechanism that may be responsible for lowering blood glucose levels in vivo (tested so far in the mouse model of diabetes) [47]. Both of these effects are integral components of the biological activity of *S. aucuparia* fruits and could explain their traditional usage in diabetes management and prevention of diabetic complications associated with oxidative stress [5]. Therefore, the ^•^OH scavenging and α-glucosidase inhibition were selected as activity models for assessing the variability in the biological effectiveness of rowanberries.

**Figure 4:**
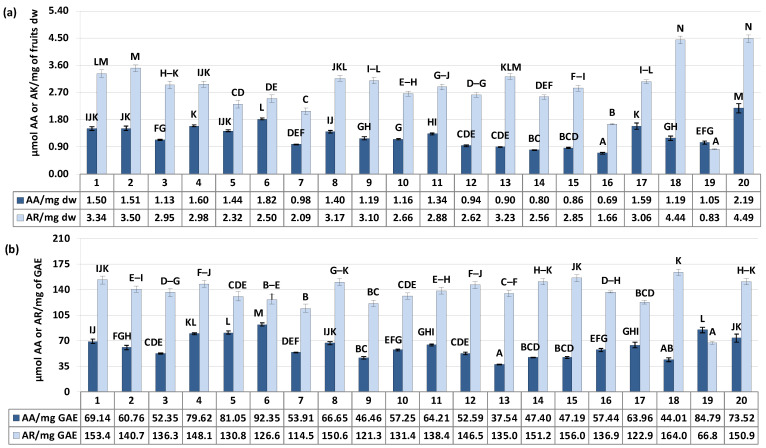
α-Glucosidase inhibition and scavenging activity against ^•^OH radical of the *S. aucuparia* fruits. Data expressed in terms of acarbose (AR, for the α-glucosidase assay) and ascorbic acid (AA, for the ^•^OH scavenging assay) equivalents per (**a**) fruits dry weight (μmol AR/mg dw or μmol AA/mg dw) or (**b**) per polyphenols weight (μmol AR/mg GAE or μmol AA/mg GAE) based on the IC_50_/SC_50_ values. Acarbose IC_50_ 176.4 ± 7.6 μg/mL (1.55 μmol/mg), ascorbic acid SC_50_ 155.5 ± 3.3 μg/mL (5.68 μmol/mg). Error bars indicate SD values (*n* = 3). Values on particular charts labelled with the same letter (A–N) did not differ significantly at α = 0.05.

As illustrated in Figure 4a, the SC_50_/IC_50_ values (effective scavenging/inhibitory concentrations) expressed in ascorbic acid (AA) or acarbose (AR) equivalents ranged from 0.69 to 2.19 μmol AA/mg and 0.83 to 4.49 μmol AR/mg of fruits dry weight for ^•^OH scavenging and α-glucosidase inhibitory activity, respectively, with the variation in samples (CV) at 29.61% and 28.85%. Considering the above results of polyphenols variability, these differences in activity may be the result of distinction in samples composition. Indeed, in accordance with our previous research [5] and literature data [6,46], the responses in the selected assays were dependent on phenolic content, showing a significant correlation with the total phenolic and total proanthocyanidin levels (*r* = 0.5890, *p* = 0.006 for TPC/^•^OH and *r* = 0.5547, *p* = 0.011 for TPA/^•^OH, in AA equivalents; *r* = 0.9167, *p* = 0.000 for TPC/α-glucosidase and *r* = 0.9305, *p* = 0.000 for TPA/α-glucosidase, in AR equivalents). However, it is known that the polyphenolsic content alone is not the sole determinant influencing plants activity. Equally important, might be the proportion between different constituents. As previously reported, the activity of flavonols, flavan-3-ols and caffeoylquinic acids against radicals can be synergistic, but this synergy strongly depends on their relative ratios [48]. 

Therefore, the last subject to investigate is to determine the impact of chemical composition variability on biological properties of rowanberries, and to what extent should quality control measures be implemented for fruit products to ensure their value?

### 3.4. The Selection of Variability Markers of Rowanberries Composition and Activity: Chemometric Analysis

To better understand the variability of phenolic composition, the relationship between individual components, and their influence on the biological activity of *S. aucuparia* fruits, a deeper analysis using chemometric tools (HCA and PCA) was performed. 

**Figure 5:**
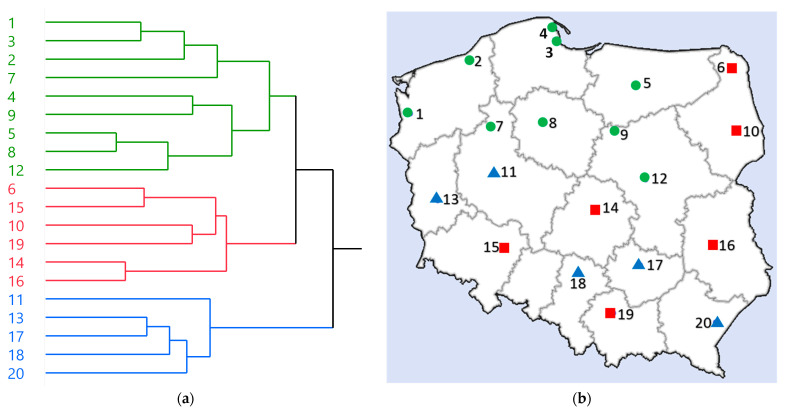
HCA analysis results. (**a**) Dendrogram obtained by HCA; (**b**) A map of Poland showing the origin of samples divided into groups based on HCA; green cluster, circle (samples nos. 1, 2, 3, 4, 5, 7, 8, 9, 12); red cluster, square (samples nos. 6, 10, 14, 15, 16, 19); blue cluster, triangle (samples nos. 11, 13, 17, 18, 20).

First, the HCA was performed based on the quantification results of 19 major phenolics (compounds that were within the limit of quantification of HPLC-PDA), TPA levels, and ^•^OH scavenging and α-glucosidase inhibition parameters (22 variables in total). As a result, the samples were categorized into three groups: these obtained specifically from northern Poland (green cluster; circle), these from eastern and also partially from southern Poland (red cluster; square), and these partially from southern and western Poland (blue cluster; triangle) (Figure 5). The results indicate that while there are overlaps in geographical locations between blue and red clusters, the chemical composition and biological properties of rowanberries in Poland may be influenced, to some extent, by their geographic origin.

**Figure 6:**
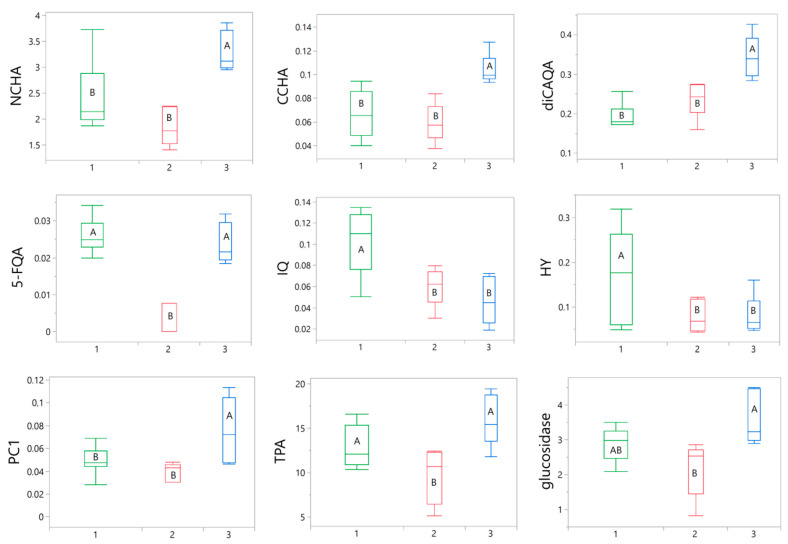
Box plots illustrating the variations in nine selected parameters between green (1), red (2), and blue (3) clusters. The width of the boxes depends on the number of samples in particular cluster. The statistical significance of differences between the mean values was determined using a one-way ANOVA, followed by the post hoc Fisher’s LSD for multiple comparison. Within particular chart means for boxes labeled with the same letter (A, B) did not differ significantly at α = 0.05. IQ, isoquercitrin; HY, hyperoside; CCHA, 4-*O*-caffeoylquinic acid; NCHA, 3-*O*-caffeoylquinic acid; 5-FQA, 5-feruloylquinic acid; diCAQA, 3,5-*O*-dicaffeoylquinic acid; PC1, procyanidin C1; TPA, total proanthocyanidins; glucosidase, α-glucosidase inhibitory activity.

Subsequently, an inter-cluster ANOVA analysis was conducted to identify the parameters influencing the categorization. Out of all the tested variables, only nine were found to significantly contribute to the differentiation of the samples. These variables included 3-*O*-caffeoylquinic acid (NCHA), 4-*O*-caffeoylquinic acid (CCHA), 3,5-*O*-dicaffeoylquinic acid (diCAQA), 5-feruloylqunic acid (5-FQA), HY, IQ, procyanidin C1 (PC1), TPA, and α-glucosidase inhibition. The variations in these parameters among the groups are presented in Figure 6.

**Figure 7:**
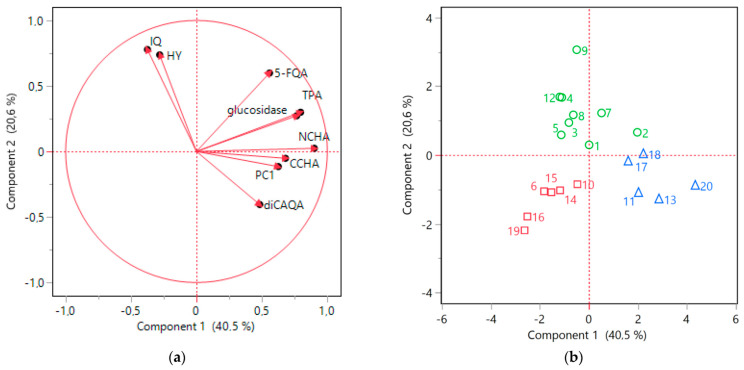
PCA analysis results. (**a**) Loading plot; (**b**) score plot for 9 investigated variables (isoquercitrin, IQ; hyperoside, HY; 4-*O*-caffeoylquinic acid, CCHA; 3-*O*-caffeoylquinic acid, NCHA; 5-feruloylquinic acid, 5-FQA; 3,5-*O*-dicaffeoylquinic acid, diCAQA; procyanidin C1, PC1; total proanthocyanidins, TPA; and α-glucosidase inhibitory activity). Green cluster, circle (samples nos. 1, 2, 3, 4, 5, 7, 8, 9, 12); red cluster, square (samples nos. 6, 10, 14, 15, 16, 19); blue cluster, triangle (samples nos. 11, 13, 17, 18, 20).

The nine selected parameters were subsequently subjected to PCA to explore the interrelationships between the variables. The first two principal components (Component 1 and Component 2) collectively accounted for over 60% of variability in the data. The resulting scores plot was in accordance with the HCA outcomes, demonstrating a clear distinction between the clusters identified earlier (Figure 7b). Moreover, the collective findings from ANOVA and PCA provided a more detailed characterization of these specific clusters. 

The blue cluster was distinguished by high Component 1 values, which were significantly positively influenced by elevated levels of phenolic acids and procyanidins included in the analysis (Figure 7b). Indeed, the samples belonging to blue cluster contained on average significantly higher levels of NCHA, CCHA, diCAQA, and PC1 (Figure 6). Furthermore, the loading plot (Figure 7a) reaffirmed the substantial correlation previously observed between TPA levels and α-glucosidase inhibitory activity. The potency of α-glucosidase inhibition also positively loaded onto Component 1, with the samples in the blue cluster displaying the highest activity in this regard. 

All the samples within the green cluster exhibited positive Component 2 scores, primarily associated with the notable presence of IQ and HY (Figure 7a,b), which were found to be, on average, more abundant in this cluster compared to the others (Figure 6). A robust correlation between the levels of the two flavonols was also evident (Figure 7a). Although the green cluster had lower contents of NCHA, CCHA, and diCAQA compared to the blue cluster, no statistically significant differences were observed between the two clusters in terms α-glucosidase inhibitory activity. This suggest that high levels of flavonoids might partly compensate for the lower phenolic acids levels in the green cluster. 

The red cluster comprised samples with negative scores for both Component 1 and Component 2 (Figure 7b). This was reflected in lower flavonoid (IQ and HY) content compared to the samples of the green cluster and a lower phenolic acid content compared to the samples from blue cluster (Figure 6). Additionally, the red cluster exhibited reduced TPA and α-glucosidase inhibitory activity compared to both of the other clusters. Thus, samples grouped in this cluster might be considered of inferior quality, particularly for functional applications aimed at inhibiting sugar absorption. 

Considering the conducted chemometric analysis, it appears that the total proanthocyanidin content (TPA) along with seven specific individual phenolic compounds could serve as valuable variability markers of *S. aucuparia* fruits. The individual constituents comprise two flavonoids (IQ, HY), four phenolic acids (NCHA, CCHA, diCAQA, 5-FQA), and one proanthocyanidin (PC1). These parameters have been proven effective in categorizing the tested samples into distinct groups that differ in composition. 

Some of these differences might have attributed to the geographic origin and associated climatic conditions. The samples belonging to the green cluster, originating from the northern part of Poland, might have benefited from a milder climate influenced by the proximity of the Baltic Sea. This region experiences cooler summers and relatively warmer winters compared to the rest of the country [49]. Such climatic conditions appear to favor the biosynthesis of quercetin derivatives, particularly its simple monoglycosides such as IQ and HY. Similar pattern of flavonoids biosynthesis was previously reported for, e.g., *S. domestica* leaves—samples collected from the seaside location in Croatia accumulated even up to several dozen times higher amount of IQ and HY than samples from the central part of Poland [35].

The eastern region of Poland is under the influence of Eurasian continental air masses, resulting in harsher winters and hotter summers. Furthermore, the eastern region’s growing season tends to be shorter compared to the western part of the country [49], which may limit the production of active metabolism and account for the comparatively poorer results obtained for samples within the red cluster sourced from the eastern boundary. The maritime air reaching the western part of Poland seems, in turn, to promote the production of active metabolites, especially proanthocyanidins and phenolic acids. Nonetheless, some variations in composition (especially for samples from southern part of Poland) could not be solely attributed to the broad geographic origin factors and might be influenced by more localized factors, such as soil conditions. Moreover, some factors may have a dual effect on the plants metabolites production, depending on their intensity and duration. For example, it was reported that while moderate drought stress promote the polyphenols biosynthesis, the prolonged drought leads to a reduction in fruits mass and the content of polyphenols in the, e.g., *S. domestica* fruits [34]. This may also partly explain the intertwining of blue and red clusters, where different air mass and other conditions can affect the plant material growth.

The observed differences in the levels of selected variability markers were also associated with the biological properties of the fruits, especially their α-glucosidase inhibitory potential. To achieve the highest activity in this regard, it is advisable to ensure high levels of TPA and phenolic acids, particularly the predominant NCHA. Moreover, the plant material from northeastern parts of Poland appears to be more suitable, on average, for functional applications in this context. As for ^•^OH scavenging activity, this study did not identify clear markers of antioxidant effectiveness, as there were no statistically significant inter-cluster differences. This could be attributed to the complex interactions between different constituents (additive and/or synergistic) [50]. However, it is worth noting that sample 6 in the red cluster displayed one of the highest ^•^OH neutralizing potential, making it an outlier among the other samples in this cluster, which exhibited below-average scavenging activity. In fact, after excluding sample 6 from the analysis, red cluster had significantly lower antioxidant capacity compared to two other cluster. Therefore, in our opinion, the selected phenolics can still be considered as markers of antioxidant activity suitable for the quality control of rowanberries, and they should be suitable for most of the samples. 

Additionally, considering the impact of polyphenol proportions on antioxidant synergy [43], the distinctive ratio of TFL, TCHA, and TPA (1:5:15) in sample 6 might be worth further studies to determine if this specific composition might be the cause of the unexpectedly high scavenging potential of the sample. 

## 4. Conclusions

This study contributes to the current understanding of the geographical variability in the phenolic composition and biological activity of *S. aucuparia* fruits. The results indicate that the overall variation in rowanberries in Poland is generally low to moderate for most of the tested factors, with the exception of flavonoid content. The variability appears to be impacted at least partially by geographic location, which might help explain the conflicting data reported on *S. aucuparia* fruit composition from different countries or regions [5,10,12,13,14,15,16,17]. Moreover, it highlights the importance of rigorous quality control for rowanberries. To address this, we employed statistical methods to identify eight parameters that are optimal for the effective quality control of rowanberries. By evaluating the content of these selected markers, we were able to differentiate the tested fruits into distinct groups based on their composition and classify the samples as more or less valuable plant materials for functional applications. Importantly, this approach yielded results comparable to those obtained through full phytochemical profiling and its efficiency makes it suitable for routine testing, saving both time and cost.

## Figures and Tables

**Table 1:** The locations of the fruit samples collection and geographical conditions during growing season (IV–XI 2019).

Sample No.	Location	Voucher Specimen	Average Air Temperature (°C) *	Average Air Humidity (%) *
1	Szczecin (14°34′ E, 53°26′ N), NH	KFG/HB/11019/SAUC/F/ZM	16.2	69
2	Koszalin (16°11′ E, 54°11′ N), NH	KFG/HB/12019/SAUC/F/ZM	15.4	72
3	Gdynia (18°32′ E, 54°32′ N), NH	KFG/HB/13019/SAUC/F/ZM	15.3	74
4	Wladyslawowo (18°24′ E, 54°48′ N), NH	KFG/HB/14019/SAUC/F/ZM	14.9	77
5	Olsztyn (16°07′ E, 52°07′ N), NH	KFG/HB/15019/SAUC/F/ZM	15.3	69
6	Suwalki (22°56′ E, 54°06′ N), NH	KFG/HB/16019/SAUC/F/ZM	14.9	67
7	Pila (16°45′ E, 53°10′ N), NH	KFG/HB/17019/SAUC/F/ZM	16.4	66
8	Bydgoszcz (18°00′ E, 53°07′ N), CT, Garden of medicinal and cosmetic plants, Collegium Medicum	KFG/HB/18019/SAUC/F/ZM	16.4	66
9	Zielun (19°51′ E, 53°10′ N), NH	KFG/HB/19019/SAUC/F/ZM	15.9	68
10	Bialystok (23°10′ E, 53°08′ N), NH	KFG/HB/20019/SAUC/F/ZM	15.1	71
11	Poznan (16°55′ E, 52°25′ N), NH	KFG/HB/21019/SAUC/F/ZM	17.0	63
12	Smoszewo (20°30′ E, 52°26′ N), NH	KFG/HB/22019/SAUC/F/ZM	17.1	64
13	Zielona Gora (15°30′ E, 51°56′ N), NH	KFG/HB/23019/SAUC/F/ZM	16.8	63
14	Lodz (19°24′ E, 51°48′ N), NH	KFG/HB/24019/SAUC/F/ZM	16.3	66
15	Wroclaw (17°02′ E, 51°07′ N), NH	KFG/HB/25019/SAUC/F/ZM	17.2	66
16	Lublin (22°34′ E, 51°14′ N), NH	KFG/HB/26019/SAUC/F/ZM	15.9	70
17	Kielce (20°36′ E, 50°52′ N), CT, Geopark—Botanical Garden	KFG/HB/27019/SAUC/F/ZM	15.9	72
18	Czestochowa (19°07′ E, 50°48′ N), NH	KFG/HB/28019/SAUC/F/ZM	16.3	69
19	Krakow (19°57′ E, 50°03′ N), NH	KFG/HB/29019/SAUC/F/ZM	15.9	71
20	Bolestraszyce (22°51′ E, 49°49′ N), CT, Arboretum and Department of Physiography	KFG/HB/30019/SAUC/F/ZM	16.4	73

NH, natural habitat; CT, cultivation. * Data according to the report of the Institute of Meteorology and Water Management-National Research Institute [24], based on measurements made by the nearest meteorological stations.

**Table 2:** UHPLC-PDA-ESI-MS^n^ identification data of polyphenols detected in the *S. aucuparia* fruits.

	Analyte	*R_t_* (min)	UV λ_max_ (nm)	[M–H]^−^ (*m*/*z*)	MS/MS Fragmentation	Sample No.
1	caffeic acid derivative	4.9	320	407	389(100), 295(21), 277(19), 179(36)	all samples
2	caffeic acid derivative	5.6	320	407	389(100), 295(22), 277(33), 235(4), 179(48)	all samples
3	3-*O*-caffeoylquinic acid (neochlorogenic acid, NCHA) ^a^	6.3	325	353	191(100), 179(48), 135(2)	all samples
4	caffeoylquinic acid derivative	8.5	325	517	353(100), 191(47)	all samples
5	caffeoylquinic acid derivative	9.2	325	517	353(100), 335(50), 191(38), 179(2)	all samples
6	*p*-coumaroylquinic acid isomer	9.4	310	337	191(9), 163(100)	all samples
7	caffeic acid derivative	10.1	320	629	471(86), 359(9), 291(100), 179(9)	all samples
8	vanilic acid hexose conjugate	10.3	284	329	283(15), 167(100)	all samples
9	amygdalin ^a^	10.8	210	456	456(100), 323(5), 323(100) ^b^, 221(5) ^b^	all samples
10	5-*O*-caffeoylquinic acid (chlorogenic acid, CHA) ^a^	11.3	325	353	191(100), 179(4)	all samples
11	4-*O*-caffeoylquinic acid (cryptochlorogenic acid, CCHA)^a^	12.6	325	353	191(100), 179(57), 173(87), 215(3)	all samples
12	procyanidin trimer B-type	13.3	279	865	713(36), 695(100), 577(67), 543(38), 407(32), 287(25), 695(100) ^b^,543(62) ^b^, 407(100) ^b^	1, 3, 4, 16, 17, 20
13	vanilic acid hexose conjugate	14.5	284	329	269(14), 191(100), 167(28)	all samples
14	1-*O*-caffeoylquinic acid (1-CHA)	15.1	325	353	215(31), 191(100), 179(5)	all samples
15	procyanidin B-2 ^a^	15.2	279	577	451(17), 425(100), 407(32), 289(11)	all samples
16	(-)-epicatechin ^a^	16.5	279	289	245(100), 205(25)	all samples
17	5-caffeoylshikimic acid	16.5	325	335	291(10), 179(100), 161(34), 135(25)	all samples
18	3-caffeoylshikimic acid	17.8	325	335	179(100), 161(2), 135(17)	all samples
19	procyanidin tetramer B-type	18.3	279	1153	1027(100), 983(42), 863(44), 739(40), 577(38), 449(18)	2, 3, 4, 5, 7, 8, 10, 11, 12, 13, 15, 16, 17, 18, 19, 20
20	3-*O*-feruloylquinic acid	19.7	325	367	335(12), 193(12), 161(100), 135(22)	all samples
21	caffeoylshikimic acid	20.0	325	335	179(11), 161(100), 135(73)	all samples
22	procyanidin C-1 (PC1) ^a^	20.8	279	865	739(47), 695(100), 577(72), 451(36), 407(53), 287(22)	all samples
23	quercetin 3-*O*-*β*-sophoroside (SQ) ^a^	21.8	350	625	579(4), 463(27), 445(42), 355(12), 301(100) ^b^	all samples
24	5-*O*-feruloylquinic acid (5-FQA)	22.3	325	367	335(3), 191(31), 179(100), 161(9), 135(32)	all samples
25	quercetin *O*-dihexoside (diHQ)	22.6	255, 352	625	579(16), 463(35), 445(53), 355(12), 301(100) ^b^, 151(45) ^b^	all samples
26	procyanidin tetramer B-type	23.2	279	1153	1027(62), 983(75), 863(73), 739(45), 575(50), 449(13)	all samples
27	quercetin-hexoside-pentoside	24.8	253,353	595	463(20), 343(22) ^b^, 301(100) ^b^	1, 2, 4, 6, 7, 9, 10, 11, 12, 14, 16, 17, 19, 20
28	kaempferol *O*-dihexoside	25.2	266, 343	609	429(38), 285(100)	2, 4, 6, 7, 9, 11, 13, 14, 15, 17, 19, 20
29	procyanidin trimer B-type	26.0	279	865	713(35), 695(100), 577(13), 289(5), 695(100) ^b^, 543(82) ^b^, 425(74) ^b^, 407(53) ^b^	all samples
30	cinchonain I isomer	26.5	283	451	341(100), 217(3)	2, 3, 4, 5, 7, 8, 9, 10, 11, 12, 13, 14, 15, 16, 17, 18, 19,20
31	quercetin 3-*O*-*β*-D-galactoside (hyperoside, HY) ^a^	27.5	254, 353	463	301(100)	all samples
32	quercetin 3-*O*-*β*-D-(6′′-*O*-α-L-rhamnosyl)-glucoside (rutin, RT) ^a^	28.0	256, 355	609	301(100)	all samples
33	quercetin 3-*O*-*β*-D-glucoside (isoquercitrin, IQ) ^a^	28.7	256, 353	463	301(100)	all samples
34	procyanidin tetramer B-type	29.3	279	1153	983(40), 863(35), 739(18), 577(16), 507(13), 449(25)	all samples
35	procyanidin tetramer B-type	30.6	279	1153	863(63), 771(59), 739(37), 577(100), 451(17), 407(18)	all samples
36	kaempferol-hexoside	30.8	265, 350	447	419(4), 327(20), 285(100)	4, 5, 7, 8, 9, 12, 13, 14, 17
37	quercetin-malonyl-hexoside	32.3	255,355	549	463(41), 301(100)	1, 2, 3, 4, 5, 6, 7, 9, 10, 12, 14, 16, 17, 18, 19, 20
38	kaempferol-hexoside	32.7	275, 350	447	327(14), 285(100)	1, 2, 3, 4, 5, 6, 7, 8, 9, 11, 12, 13, 14, 15, 16, 17, 18, 19
39	cinchonain I isomer	32.9	280	451	341(100)	1, 3, 5, 6, 10, 14, 15, 16, 17, 18, 20
40	3,5-*O*-dicaffeoylquinic acid (diCAQA)	33.3	325	515	353(100), 191(3), 179(3), 191(100) ^b^, 179(23) ^b^	all samples
41	procyanidin tetramer B-type	34.0	279	1153	863(52), 739(27), 577(67), 407(16)	all samples
42	kaempferol-acetyl-hexoside	35.7	275, 350	489	447(9), 327(7), 285(100)	4, 7, 9, 12, 14, 17
43	kaempferol-acetyl-hexoside	37.2	275, 350	489	285(100)	1, 2, 3, 4, 5, 6, 7, 9, 12, 14, 16, 17, 18
44	cinchonain I isomer	40.6	280	451	341(100), 289(5)	all samples
45	quercetin (QU) ^a^	43.8	268, 364	301	301(100)	4, 5, 7, 9, 10, 12, 14, 16, 17, 20
46	quercetin-glucuronide-hexoside	44.7	255, 355	639	463(100), 301(33)	1, 6, 17

R_t,_ retention time. UV λ_max_, absorbance maxima in PDA spectra. [M − H]^−^, pseudomolecular ions in MS spectra recorded in a negative mode. MS^2^, secondary ions (the underlined ions were subjected to MS^3^ fragmentation). Intensities of particular ions are given in parentheses. Nomenclature of the pseudodepsides of quinic acid and shikimic acid is given according to IUPAC and Clifford et al. [29,30]. ^a^ Compounds identified with authentic standards. ^b^ MS^3^ fragmentation of the underlined ions.

**Table 3:** The average contents and the coefficients of variation (CV) for phenolic groups and individual compounds present in the *S. aucuparia* fruits.

Phenolic Groups:	Means ± SD (mg/g dw)	CV (%)	Individual Compounds:	Means ± SD (mg/g dw)	CV (%)
TPC	20.75 ± 4.49	21.62	NCHA	2.47 ± 0.76	30.79
TPH	8.01 ± 1.64	20.44	CHA	3.90 ± 1.07	27.46
THCA	7.05 ± 1.52	21.51	CCHA	0.08 ± 0.03	37.51
TCHA	6.83 ± 1.48	21.63	1-CHA	0.15 ± 0.04	27.48
TCFA	0.22 ± 0.06	25.62	diCAQA	0.23 ± 0.09	38.94
TFL	0.90 ± 0.39	43.59	5-FQA	0.02 ± 0.01	63.39
TPA	12.81 ± 3.54	27.63	PC1	0.05 ± 0.02	44.97
TLPA	0.06 ± 0.02	36.30	RT	0.06 ± 0.04	75.87
			SQ	0.41 ± 0.22	52.44
			diHQ	0.13 ± 0.06	47.27
			HY	0.13 ± 0.09	68.15
			IQ	0.07 ± 0.03	45.48

TPC, total phenolic content in gallic acid equivalents (GAE) determined by the Folin–Ciocalteu assay; TPH, total phenolic content determined by RP-HPLC-PDA; THCA, total content of hydroxycinnamic acid derivatives (TCHA + TCFA); TCHA, total content of mono- and dicaffeoylqunic acids isomers; TCFA, total content of phenolic acids derivatives other than TCHA; TFL, total content of flavonoids; TPA, total proanthocyanidin content in cyanidin chloride equivalents (CyE) determined by the *n*-butanol/HCl assay; TLPA, total content of proanthocyanidin determined by RP-HPLC-PDA; NCHA, 3-*O*-caffeoylquinic acid; CHA, 5-*O*-caffeoylquinic acid; CCHA, 4-*O*-caffeoylquinic acid; 1-CHA, 1-*O*-caffeoylquinic acid; diCAQA, 3,5-*O*-dicaffeoylquinic acid; 5-FQA, 5-ferulquinin acid; PC1, procyanidin C1; RT, rutin; SQ, quercetin 3-*O*-β-sophoroside; diHQ, quercetin *O*-dihexoside; HY, hyperoside; IQ, isoquercitrin.

## Data Availability

Data are contained within this article.

## Supplementary Materials

The following supporting information can be downloaded at: https://www.mdpi.com/article/10.3390/antiox12111967/s1, Table S1: Quantitative profile of individual compounds in the *S. aucuparia* fruits (mg/g fruits dw). Table S2: Quantitative profile of groups of compounds in the *S. aucuparia* fruits (mg/g fruits dw).
