# Supplementary material for: Chemometrics-Driven Variability Evaluation of Phenolic Composition, Antioxidant Capacity, and α-Glucosidase Inhibition of Sorbus aucuparia L. Fruits from Poland: Identification of Variability Markers for Plant Material Valorization
Source: Antioxidants (Basel). 2023 Nov 5;12(11):1967. doi: 10.3390/antiox12111967 (PMC10668990; doi:10.3390/antiox12111967)
Supplement: Supplementary file 1 [file antioxidants-12-01967-s001.zip › antioxidants-2686964-supplementary.pdf]

Table S1. Quantitative profile of individual compounds in the *S. aucuparia* fruits (mg/g fruits dw).

Sample	NCHA	CHA	CCHA	1-CHA	diCAQ	5-FQA	PC1	RT	SQ	diHQ	HY	IQ
1	2.2413 ± 0.0117^{EF}	1.8572 ± 0.0107^{A}	0.0516 ± 0.0020^{CD}	0.1071 ± 0.0010^{BCD}	0.1798 ± 0.0013^{DEF}	0.0250 ± 0.0001^{G}	0.0427 ± 0.0018^{BC}	0.0145 ± 0.0004^{B}	0.2625 ± 0.0016^{D}	0.1099 ± 0.0005^{G}	0.0492 ± 0.0005^{B}	0.0735 ± 0.0024^{FG}
2	3.7256 ± 0.0448^{J}	2.8055 ± 0.0330^{C}	0.0945 ± 0.0029^{I}	0.1184 ± 0.0017^{D}	0.1106 ± 0.0013^{B}	0.0342 ± 0.0007^{J}	0.0546 ± 0.0003^{CDE}	0.0095 ± 0.0001^{A}	0.4904 ± 0.0069^{H}	0.1066 ± 0.0005^{G}	0.0637 ± 0.0026^{D}	0.0505 ± 0.0023^{CD}
3	1.8836 ± 0.0245^{C}	3.2194 ± 0.0487^{E}	0.0456 ± 0.0014B^{C}	0.1873 ± 0.0017^{H}	0.1813 ± 0.0013^{EF}	0.0229 ± 0.0009^{EF}	0.0477 ± 0.0005^{CD}	0.0894 ± 0.0023^{K}	0.1528 ± 0.0036^{B}	0.1631 ± 0.0033^{I}	0.1231 ± 0.0042^{G}	0.1228 ± 0.0018^{J}
4	1.8660 ± 0.0033^{C}	3.8219 ± 0.0097^{F}	0.0403 ± 0.0004^{AB}	0.1675 ± 0.0063^{FG}	0.1765 ± 0.0008^{DE}	0.0249 ± 0.0007^{G}	0.0689 ± 0.0020^{EF}	0.1624 ± 0.0021^{O}	0.7775 ± 0.0009^{N}	0.2265 ± 0.0019^{L}	0.2327 ± 0.0014^{K}	0.1099 ± 0.0013^{I}
5	2.1224 ± 0.0232^{D}	4.0293 ± 0.0383^{G}	0.0690 ± 0.0006^{FG}	0.1680 ± 0.0040^{FG}	0.1882 ± 0.0008^{F}	0.0229 ± 0.0004^{EF}	0.0461 ± 0.0011^{CD}	0.0704 ± 0.0006^{I}	0.2235 ± 0.0031^{C}	0.0617 ± 0.0013^{B}	0.1758 ± 0.0016^{J}	0.0840 ± 0.0007^{H}
6	1.3977 ± 0.0240^{A}	2.7384 ± 0.0436^{C}	0.0373 ± 0.0009^{A}	0.1109 ± 0.0021^{CD}	0.2558 ± 0.0039^{I}	< LOQ^{A}	0.0423 ± 0.0007^{BC}	0.0542 ± 0.0006^{H}	0.3526 ± 0.0047^{E}	0.2081 ± 0.0038^{K}	0.0702 ± 0.0003^{E}	0.0794 ± 0.0006^{H}
7	3.5135 ± 0.0641^{I}	2.7591 ± 0.0508^{C}	0.1425 ± 0.0025^{K}	0.0998 ± 0.0043^{BC}	0.0233 ± 0.0004^{A}	0.0233 ± 0.0004^{F}	0.0610 ± 0.0010^{DEF}	0.1414 ± 0.0020^{N}	0.5057 ± 0.0079^{HI}	0.1904 ± 0.0023^{J}	0.1516 ± 0.0026^{H}	0.1350 ± 0.0039^{K}
8	2.1450 ± 0.0172^{DE}	4.7633 ± 0.0348^{I}	0.0776 ± 0.0020^{H}	0.2000 ± 0.0047^{I}	0.1718 ± 0.0012^{D}	0.0200 ± 0.0003^{C}	0.0460 ± 0.0021^{CD}	0.0127 ± 0.0005^{AB}	0.0630 ± 0.0008^{A}	< LOQ^{A}	0.2445 ± 0.0022^{L}	0.0786 ± 0.0010^{GH}
9	2.2507 ± 0.0125^{EF}	5.4233 ± 0.0284^{L}	0.0656 ± 0.0001^{FG}	0.2291 ± 0.0005^{K}	0.2373 ± 0.0011^{H}	0.0337 ± 0.0003^{J}	0.0283 ± 0.0013^{B}	0.1130 ± 0.0005^{M}	0.7292 ± 0.0021^{M}	0.1575 ± 0.0010^{I}	0.3187 ± 0.0002^{N}	0.1328 ± 0.0009^{K}
10	2.2547 ± 0.0358^{F}	4.4551 ± 0.0613^{H}	0.0577 ± 0.0007^{DE}	0.2149 ± 0.0039^{J}	0.2756 ± 0.0036^{JK}	0.0306 ± 0.0003^{I}	< LOQ^{A}	0.0237 ± 0.0005^{D}	0.2660 ± 0.0037^{D}	0.1064 ± 0.0022^{G}	0.0439 ± 0.0012^{A}	0.0301 ± 0.0011^{B}

11	3.5653 ± 0.0768^{I}	4.8771 ± 0.1126^{I}	0.1275 ± 0.0034^{J}	0.1418 ± 0.0050^{E}	0.2836 ± 0.0055^{K}	0.0318 ± 0.0004^{I}	0.0489 ± 0.0012^{CD}	0.0203 ± 0.0006^{C}	0.7105 ± 0.0184^{L}	0.0999 ± 0.0036^{F}	0.0654 ± 0.0010^{DE}	0.0187 ± 0.0006^{A}
12	2.0963 ± 0.0186^{D}	5.0332 ± 0.0460^{J}	0.0627 ± 0.0008^{EF}	0.1765 ± 0.0073^{GH}	0.2573 ± 0.0027^{I}	0.0251 ± 0.0002^{G}	0.0474 ± 0.0014^{CD}	0.0933 ± 0.0007^{L}	0.4250 ± 0.0024^{G}	0.0836 ± 0.0020^{D}	0.2795 ± 0.0017^{M}	0.1186 ± 0.0010^{J}
13	3.1189 ± 0.0839^{H}	4.4065 ± 0.1140^{H}	0.0997 ± 0.0048^{I}	0.1461 ± 0.0048^{E}	0.4274 ± 0.0076^{O}	0.0271 ± 0.0012^{H}	0.0959 ± 0.0020^{G}	0.0340 ± 0.0005^{EF}	0.3415 ± 0.0072^{E}	0.1452 ± 0.0022^{H}	0.0560 ± 0.0013^{C}	0.0447 ± 0.0011^{C}
14	2.2372 ± 0.0156^{EF}	3.0591 ± 0.0209^{D}	0.0837 ± 0.0014^{H}	0.1090 ± 0.0018^{BCD}	0.2305 ± 0.0009^{H}	< LOQ^{A}	0.0436 ± 0.0008^{BC}	0.0318 ± 0.0003^{E}	0.4054 ± 0.0029^{F}	0.0916 ± 0.0012^{E}	0.1159 ± 0.0006^{F}	0.0719 ± 0.0004^{F}
15	1.5717 ± 0.0096^{B}	4.4021 ± 0.0216^{H}	0.0565 ± 0.0007^{DE}	0.1442 ± 0.0027^{E}	0.1608 ± 0.0009^{C}	< LOQ^{A}	0.0409 ± 0.0009^{BC}	0.0855 ± 0.0004^{J}	0.3484 ± 0.0017^{E}	0.2412 ± 0.0005^{M}	0.0472 ± 0.0003^{AB}	0.0617 ± 0.0028^{E}
16	1.6726 ± 0.0049^{B}	2.4859 ± 0.0035^{B}	0.0498 ± 0.0021^{C}	0.0868 ± 0.0024^{A}	0.2177 ± 0.0005^{G}	< LOQ^{A}	0.0478 ± 0.0012^{CD}	0.0532 ± 0.0005^{H}	0.1704 ± 0.0004^{B}	0.0689 ± 0.0007^{C}	0.0671 ± 0.0002^{DE}	0.0628 ± 0.0003^{E}
17	2.9579 ± 0.0165^{G}	5.2530 ± 0.0450^{K}	0.0937 ± 0.0003^{I}	0.1589 ± 0.0025^{F}	0.3564 ± 0.0015^{N}	0.0206 ± 0.0004^{CD}	0.0723 ± 0.0027^{F}	0.0428 ± 0.0006^{G}	0.6544 ± 0.0050^{J}	0.1494 ± 0.0007^{H}	0.1603 ± 0.0010^{I}	0.0659 ± 0.0004^{E}
18	3.0171 ± 0.0177^{GH}	5.4079 ± 0.0366^{L}	0.0989 ± 0.0003^{I}	0.1406 ± 0.0025^{E}	0.3090 ± 0.0031^{L}	0.0184 ± 0.0001^{B}	0.0461 ± 0.0004^{CD}	0.0228 ± 0.0006^{CD}	0.1606 ± 0.0020^{B}	0.0639 ± 0.0003^{BC}	0.0672 ± 0.0011^{DE}	0.0726 ± 0.0047^{F}
19	1.8835 ± 0.0140^{C}	3.3703 ± 0.0224^{E}	0.0695 ± 0.0003^{G}	0.0986 ± 0.0025^{B}	0.2739 ± 0.0010^{J}	< LOQ^{A}	0.0449 ± 0.0011^{CD}	0.0371 ± 0.0006^{F}	0.6917 ± 0.0059^{K}	0.1617 ± 0.0036^{I}	0.1214 ± 0.0020^{G}	0.0507 ± 0.0018^{D}
20	3.8660 ± 0.0133^{K}	3.7559 ± 0.0102^{F}	0.1000 ± 0.0046^{I}	0.1406 ± 0.0025^{E}	0.3393 ± 0.0016^{M}	0.0216 ± 0.0005^{DE}	0.1136 ± 0.0230^{H}	0.0418 ± 0.0009^{G}	0.5120 ± 0.0024^{I}	0.1615 ± 0.0011^{I}	0.0478 ± 0.0012^{AB}	0.0323 ± 0.0008^{B}

Results are presented as means ± SD (n = 3). For each parameter, different superscript letters indicate significant differences ($p < 0.05$). LOQ, limit of quantification (HPLC analysis); NCHA, 3-*O*-caffeoylquinic acid; CHA, 5-*O*-caffeoylquinic acid; CCHA, 4-*O*-caffeoylquinic acid; 1-CHA, 1-*O*-caffeoylquinic acid; diCAQA, 3,5-*O*-dicaffeoylquinic acid; 5-FQA, 5-ferulquinin acid; PC1, procyanidin C1; RT, rutin; SQ, quercetin 3-*O*-β-sophoroside; diHQ, quercetin *O*-dihexoside; HY, hyperoside; IQ, isoquercitrin.

Table S2. Quantitative profile of groups of compounds in the *S. aucuparia* fruits (mg/g fruits dw).

Sample	TPC	TPH	THCA	TCHA	TCFA	TFL	TPA	TLPA
1	21.7545 ± 0.5879^{F}	5.2512 ± 0.0305^{A}	4.5931 ± 0.0258^{A}	4.4371 ± 0.0239^{A}	0.1561 ± 0.0025^{B}	0.6154 ± 0.0033^{E}	15.2069 ± 0.2777^{G}	0.0427 ± 0.0018^{AB}
2	24.9164 ± 0.3177^{G}	7.9579 ± 0.0961^{G}	7.1135 ± 0.0821^{F}	6.8546 ± 0.0807^{F}	0.2589 ± 0.0017^{G}	0.7898 ± 0.0144^{H}	16.5989 ± 0.3417^{H}	0.0546 ± 0.0003^{BCDE}
3	21.6538 ± 0.6730^{F}	6.4239 ± 0.0911^{C}	5.6686 ± 0.0771^{B}	5.5172 ± 0.0752^{B}	0.1514 ± 0.0032^{B}	0.7077 ± 0.0147^{G}	14.6099 ± 0.3001^{G}	0.0477 ± 0.0005^{BC}
4	20.1049 ± 0.6163^{DEF}	7.9872 ± 0.0120^{G}	6.2566 ± 0.0053^{D}	6.0721 ± 0.0075^{C}	0.1845 ± 0.0025^{C}	1.6616 ± 0.0057^{P}	11.8068 ± 0.3214^{EF}	0.0689 ± 0.0020^{EF}
5	17.7048 ± 0.5593^{B}	7.5495 ± 0.0686^{F}	6.7744 ± 0.0606^{E}	6.5769 ± 0.0603^{DE}	0.1975 ± 0.0006^{CD}	0.7290 ± 0.0082^{G}	10.6517 ± 0.3187^{D}	0.0461 ± 0.0011^{BC}
6	19.7577 ± 0.5849^{CDE}	5.5771 ± 0.0815^{B}	4.7319 ± 0.0714^{A}	4.5399 ± 0.0712^{A}	0.1919 ± 0.0019^{C}	0.8029 ± 0.0095^{HI}	12.4759 ± 0.5308^{F}	0.0423 ± 0.0007^{AB}
7	18.2406 ± 0.5160^{BCD}	8.3463 ± 0.1463^{H}	6.9302 ± 0.1247^{EF}	6.6878 ± 0.1193^{EF}	0.2424 ± 0.0066^{F}	1.3551 ± 0.0216^{O}	11.2045 ± 0.4356^{DE}	0.0610 ± 0.0010^{CDEF}
8	21.0177 ± 0.6390^{EF}	8.0147 ± 0.0662^{G}	7.5700 ± 0.0628^{G}	7.3577 ± 0.0598^{GH}	0.2123 ± 0.0050^{DE}	0.3988 ± 0.0016^{A}	12.1098 ± 0.1754^{EF}	0.0460 ± 0.0021^{BC}
9	25.5503 ± 1.0568^{GH}	10.4224 ± 0.0503^{L}	8.5211 ± 0.0445^{H}	8.2061 ± 0.0424^{I}	0.3150 ± 0.0035^{I}	1.8730 ± 0.0046^{Q}	15.4672 ± 0.5727^{G}	0.0283 ± 0.0013^{A}
10	20.2768 ± 0.4507^{EF}	8.0799 ± 0.1145^{GH}	7.5449 ± 0.1076^{G}	7.2580 ± 0.1034^{G}	0.2869 ± 0.0043^{H}	0.4701 ± 0.0078^{B}	12.0876 ± 0.1684^{EF}	0.0650 ± 0.0009^{DEF}
11	20.8344 ± 0.9489^{EF}	10.2145 ± 0.2280^{L}	9.2509 ± 0.2058^{I}	8.9953 ± 0.2030^{J}	0.2556 ± 0.0045^{FG}	0.9147 ± 0.0230^{K}	11.7833 ± 0.5039^{EF}	0.0489 ± 0.0012^{BCD}
12	17.8909 ± 0.4772^{BC}	9.1096 ± 0.0674^{I}	7.7832 ± 0.0610^{G}	7.6259 ± 0.0615^{H}	0.1573 ± 0.0009^{B}	1.2789 ± 0.0061^{N}	10.3279 ± 0.1735^{CD}	0.0474 ± 0.0014^{BC}
13	23.9440 ± 0.6200^{G}	9.2111 ± 0.2330^{IJ}	8.4524 ± 0.2182^{H}	8.1986 ± 0.2145^{I}	0.2538 ± 0.0042^{FG}	0.6628 ± 0.0128^{F}	15.2755 ± 0.4039^{G}	0.0959 ± 0.0020^{G}
14	16.9044 ± 0.5960^{B}	6.7977 ± 0.0464^{D}	5.9032 ± 0.0410^{BC}	5.7195 ± 0.0383^{B}	0.1837 ± 0.0050^{C}	0.8509 ± 0.0062^{J}	9.3154 ± 0.0990^{C}	0.0436 ± 0.0008^{AB}
15	18.2504 ± 0.5975^{BCD}	7.3153 ± 0.0372^{EF}	6.4904 ± 0.0327^{D}	6.3353 ± 0.0316^{CD}	0.1551 ± 0.0017^{B}	0.7840 ± 0.0050^{H}	12.2140 ± 0.3628^{EF}	0.0409 ± 0.0009^{AB}
16	12.0973 ± 0.2639^{A}	5.2319 ± 0.0153^{A}	4.6335 ± 0.0158^{A}	4.5129 ± 0.0101^{A}	0.1206 ± 0.0066^{A}	0.5506 ± 0.0010^{D}	6.9237 ± 0.1045^{B}	0.0478 ± 0.0012B^{C}
17	24.9024 ± 0.5718^{G}	10.3514 ± 0.0744^{L}	9.1031 ± 0.0661^{I}	8.8198 ± 0.0641^{J}	0.2833 ± 0.0021^{H}	1.1760 ± 0.0056^{M}	15.4490 ± 0.5509^{G}	0.0723 ± 0.0027^{F}

18	27.0718 ± 0.5877 ^{H}	9.7607 ± 0.0773 ^{K}	9.2005 ± 0.0684 ^{I}	8.9736 ± 0.0581 ^{J}	0.2269 ± 0.0107 ^{E}	0.5141 ± 0.0100 ^{C}	18.0127 ± 0.4146 ^{I}	0.0461 ± 0.0004 ^{BC}
19	12.3571 ± 0.3421 ^{A}	7.0648 ± 0.0330 ^{DE}	5.9573 ± 0.0295 ^{C}	5.6958 ± 0.0351 ^{B}	0.2615 ± 0.0082 ^{G}	1.0626 ± 0.0060 ^{L}	5.1216 ± 0.2020 ^{A}	0.0449 ± 0.0011 ^{BC}
20	29.7552 ± 0.5935 ^{I}	9.4390 ± 0.0157 ^{J}	8.4969 ± 0.0389 ^{H}	8.2018 ± 0.0322 ^{I}	0.2950 ± 0.0073 ^{H}	0.8286 ± 0.0027 ^{IJ}	19.4843 ± 0.3652 ^{J}	0.1136 ± 0.0230 ^{H}

Results are presented as means ± SD (n = 3). For each parameter, different superscript letters indicate significant differences ($p < 0.05$). TPC, total phenolic content in gallic acid equivalents (GAE) determined by Folin-Ciocalteu assay; TPH, total phenolic content determined by RP-HPLC-PDA; THCA, total content of hydroxycinnamic acid derivatives (TCHA+TCFA); TCHA, total content of mono- and dicaffeoylquinic acids isomers; TCFA, total content of phenolic acids derivatives other than TCHA; TFL, total content of flavonoids; TPA, total proanthocyanidin content in cyanidin chloride equivalents (CyE) determined by *n*-butanol/HCl assay; TLPA, total content of proanthocyanidin determined by RP-HPLC-PDA.
